# Aromatic Profile Variation of Essential Oil from Dried Makwhaen Fruit and Related Species

**DOI:** 10.3390/plants10040803

**Published:** 2021-04-19

**Authors:** Trid Sriwichai, Jiratchaya Wisetkomolmat, Tonapha Pusadee, Korawan Sringarm, Kiattisak Duangmal, Shashanka K. Prasad, Bajaree Chuttong, Sarana Rose Sommano

**Affiliations:** 1 Plant Bioactive Compound Laboratory (BAC Lab), Department of Plant and Soil Sciences, Faculty of Agriculture, Chiang Mai University, Chiang Mai 50200, Thailand; trid_s@cmu.ac.th (T.S.); jiratchaya_wis@cmu.ac.th (J.W.); 2 Innovative Agriculture Research Center, Faculty of Agriculture, Chiang Mai University, Chiang Mai 50200, Thailand; tonapha.p@cmu.ac.th (T.P.); kanok70@hotmail.com (K.S.); 3 Plant Genetic Resource and Nutrition Laboratory, Department of Plant and Soil Sciences, Faculty of Agriculture, Chiang Mai University, Chiang Mai 50200, Thailand; 4 Department of Animal and Aquatic Sciences, Faculty of Agriculture, Chiang Mai University, Chiang Mai 50200, Thailand; 5 Department of Food Technology, Faculty of Science, Chulalongkorn University, Bangkok 10330, Thailand; kiattisak.d@chula.ac.th; 6 Emerging Process for Food Functionality Design Research Unit, Chulalongkorn University, Bangkok 10330, Thailand; 7 Department of Biotechnology and Bioinformatics, Faculty of Life Sciences, JSS Academy of Higher Education and Research, Mysuru 570004, India; shashankaprasad@jssuni.edu.in; 8 Meliponini and Apini Research Laboratory, Department of Entomology and Plant Pathology, Faculty of Agriculture, Chiang Mai University, Chiang Mai 50200, Thailand

**Keywords:** aromatic plant, chemical profiles, huajiao, spicy plant, taxonomical description, volatile compositions

## Abstract

The aim of this research is to evaluate the relationship between genotype, phenotype, and chemical profiles of essential oil obtained from available *Zanthoxylum* spp. Three specimens of makhwaen (MK) distributed in Northern Thailand were genetically and morphologically compared with other *Zanthoxylum* spices, known locally as huajiao (HJ) and makwoung (MKO), respectively. HJ was taxonomically confirmed as *Z. armatum* while MKO and MK were identified as *Z. rhetsa* and *Z. myriacanthum*. Genetic sequencing distributed these species into three groups accordingly to their confirmed species. Essential oil of the dried fruits from these samples was extracted and analyzed for their chemical and physical properties. Cluster analysis of their volatile compositions separated MKO and MK apart from HJ with L-limonene, terpinen-4-ol, *β*-phellandrene, and *β*-philandrene. By using odor attributes, the essential oil of MKO and MK were closely related possessing fruity, woody, and citrus aromas, while the HJ was distinctive. Overall, the phenotypic characteristic can be used to elucidate the species among makhwaen fruits of different sources. The volatile profiling was nonetheless dependent on the genotypes but makwoung and makhwaen showed similar profiles.

## 1. Introduction

Plants of the *Zanthoxylum* spp. (Rutaceae) contain oil glands that yield high amounts of essential oil with distinctive aroma [1]. Their fruits are known as spices for ethnic food particular in Asia such as those of *Z*. *piperitum* [2], *Z*. *armatum* [3], *Z*. *fagara* [4], and the essential oils extracted from the fruits and leaves are used as food additives and functional ingredients in food and pharmaceutical industries. Commonly known as makhwaen or makhan, *Z*. *myriacanthum* is grown extensively in many areas of northern Thailand viz., Pong district of Payao, Song Khwae district of Nan and in many high-altitude areas of Chiang Mai [5]. Previous studies described that *Z*. *myriacanthum* essential oil gives a unique citrus top-note followed by a woody and spice aromatic profile [4,5,6]. The analysis of the volatile compositions of *Z*. *myriacanthum* has illustrated that the main compounds are comprised of sabinene, terpinene-4-ol, and L-limonene [5,6]. Moreover, essential oils of plants in this genus also possess biological activities such as antimicrobial properties [7] antioxidant activity [8] and anti-inflammatory [9] thereby are used for medicinal purposes. With an increasing commercial need for exotic ingredients, there is, therefore demands for high-quality raw material of essential oil for food and perfumery industries. To the perfumery industry claim, the complaint made from the raw material purchaser asserted that plant morphological characteristics such as tree structure, sizes, and color of the berry clusters were variable in different sourcing regions which made the final quality of essential oil unsteady (Mrs. Anne Saget pers. Comm.). Moreover, the complexity within the species remains ambiguous as such *Z*. *myriacanthum* is often misidentified as *Z*. *limonella* [10]. Thus, there is urge commercially to truly describe plant species.

Genetic and environmental variables—i.e., growing condition, light intensity, day length, temperature, altitude, as well as their interactions—could generally influence the quantity and quality of the essential oils [11,12]. Identification of plant species and variety in the same genus can be accomplished by taxonomic description and chemical compositions [13]. However, only the use of these phenomena may not be enough to accurately describe the species. Studies on the essential oil containing plants revealed that chemical compositions and characteristics of essential oils from plants within the same genus are diverse such as those belonging to *Ocimum* spp. [14] and *Zanthoxylum* spp. [15]. The use of DNA fingerprints can therefore accomplish for the reliable identification of plant species [16].

Internal transcribed spacer DNA barcode (ITS2) detects nuclear marker of the rDNA region in nuclear genome that is useful for directly detecting reticulate phenomena. This technique has been reported to be an efficient barcode locus for plant identification [17,18] and classification by many plant species such as Indian *Berberis* [19], timber species of the mahogany family [20], and *Dendrobium* species [21]. In addition to the ITS region, RAPD analysis is an alternate method for estimating genetic diversity and relatedness in plant populations, cultivars and germplasm accessions, especially in non-model plant species. By using the markers, the technique is able to amplify DNA from dispersed polymorphic loci and thereby indirectly distinguishes small differences within the gene sequences. To draw accurate conclusion on genetic relations of plants species, it is therefore vital to combine these techniques. There is no research work to-date that fully describe genotyping differences among raw materials for makhwaen essential oil production in relation to their physical properties and aromatic profiles as compared to those of other *Zanthoxylum* species. The aim of this research, therefore, is to descriptively establish profile specification of raw materials used in makhwaen essential oil extraction industry.

## 2. Results and Discussion

### 2.1. Morphological Confirmation

The morphological descriptions of the specimens of the *Zanthoxylum* spp. known locally as huajiao (HJ), makwoung (MKO), and makwhaen (MK1-3) were documented using plant structure, thorn, leaf type, floral structures, and fresh fruit color [22]. From our data in Supplementary Table S1, the plant structure of HJ was of shrub and was different from that of MKO and MK1-3 (tree-like structure). Thorns of all specimens were either initiate on the trunk or branches. The same compound leaf type was observed in the MKO and MK1-3 (even-pinnately) which were different from the HJ (odd-pinnately compound leaf). The floral compositions were different in every species; the HJ consisted of the flower with six to nine petals, while the MKO was with four petals and MK1-3 were with five petals. Within a similar pattern, the number of anthers was different in every species, four to eight anthers for HJ, three or four anthers for MKO, and five anthers for MK1-3. The color of fresh fruit was red in HJ and MKO while MK1-3 gave greenish-red color characteristics. Fruit sizes varied from 2–3 mm of the MK1-3 to 4–5 mm of the HJ and the MKO was 5–7 mm, respectively. The three species gave brown fruit when dried with crack revealing the inner seeds. According to these specific characteristics, the scientific names of *Z*. *armatum, Z*. *rhetsa*, and *Z*. *myriacanthum* are given to HJ, MKO, and MK specimens [22,23]. To describe the verity within the same species, floral and fruit characteristics of MK1-3 were compared (Supplementary Table S2). The result confirmed that the MKs were those of *Z*. *myriacanthum* as the sepals and petals were pentamerous and male flower organs composed five stamens.

According to the results from UPGMA analysis of plant characteristics and seven samples of *Zanthoxylum* spp., MK1-3 were far distinctive from MKO and HJ. As it could be seen, MKO and MK1-3 were both tree plants while HJ was a shrub. Nonetheless, MK1-3 were detached from MKO by their floral characteristics (Figure S1).

### 2.2. ITS Sequencing Analysis

**Figure 1:**
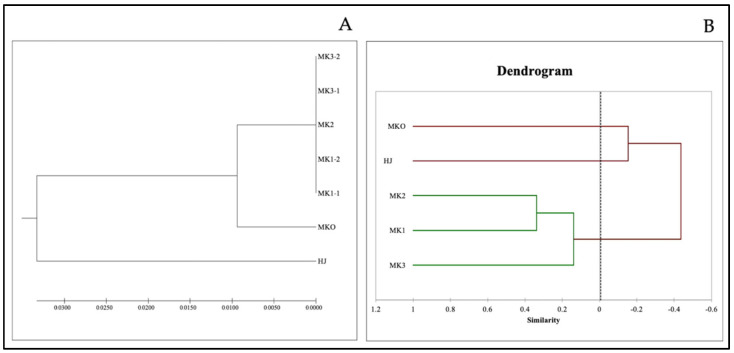
The dendrogram of *Zanthoxylum* spp. in North of Thailand; HJ (huajiao), MKO (makwoung), MK1 (makhwaen from Mae Tang district), MK2 (makhwaen from Mae Rim district), and MK3 (makhwaen from Song Kwae district) derived by UPGMA from the similarity matrix of the ITS sequence data (**A**) and from the similarity matrix based on 37 DNA bands obtained from five RAPD markers (**B**).

The aligned lengths of the ITS region (including both ITS1 and ITS2 regions) ranged from 596 bp for MK (*Z. myriacanthum*) to 600 bp for HJ (*Z. armatum*). Among the five MK sampling (two specimens from Mae Tang district: MK1-1 and MK1-2, one from Mae Rim: MK2 and two from Nan: MK3-1 and MK3-2), the ITS sequences were completely identical whereas 39 single nucleotide polymorphism were found among MK, MKO, and HJ samples. The phylogenetic relationship analysis was investigated based on the total ITS region sequences. The dendrogram showed three major clades (Figure 1A), the first formed among five MK samples from the three regions, the second consisted of MKO while the last is HJ. ITS sequence is an efficient tool for genetic identification among species however, very low efficiency for evaluation of genetic variation within the species.

### 2.3. RAPD Analysis

Only S6, S7, S9, OPA01, and OPA04 primers gave responses with the DNA thus they were used for the calculation of the unweighted pair group method with arithmetic mean (UPGMA). Result was illustrated as a dendrogram in which the samples were split into three groups: group 1—HJ, group 2—MKO, and group 3 consisting of MK1-3, as shown in Figure 1B. The dendrogram illustrated that MK1-3 were clustered as closely related species while HJ and MKO were genetically identified as separated species. Indeed, the RAPD makers revealed slight genetic variables between the *Z. myriacanthum* samples from different geographical regions. The result was correspondent with our taxonomic data described previously. In addition to the ITS sequencing, the RAPD technique was successful to determine the genetic variation of the *Zanthoxylum* spp. as well as many plants of this kind including *Z*. *hamiltonianum, Z*. *nitidum, Z*. *oxyphyllum, Z*. *rhesta, Z*. *armatum*, and *Z*. *schinifolium* [24,25,26].

### 2.4. Essential Oil Analysis

Essential oils were extracted from dried fruits of makhwaen samples from three areas (MK1, MK2, and MK3), huajiao (HJ) and makwoung (MKO) using hydro-distillation. The extraction yield varied by mean of species differentiation i.e., MK1-3 (~7%), followed by HJ (~5%) and MKO (~2%). Thirty-five volatile compounds were detected using GC-MS (Table 1). Essential oil of the MKO contained the major content of linalool (7.35 µg·mL^−1^) following by β-thujone (1.03 µg·mL^−1^) and sabinene (0.44 µg·mL^−1^), respectively. Sabinene was the key dominant substance in the *Zanthoxylum* species analyzed, except for the essential oil of the MKO. This is in agreement with other works done with plants belongs to the *Zanthoxylum* species—i.e., *Z*. *xanthoxyloïdes, Z*. *leprieurii* [27], and *Z*. *rhoifolium* [28] with sabinene and limonene that represented woody and citrus aromas [29].

The chemical profiles of the essential oils from makhwaen fruits collected from different locations were variable. The major components of all samples could be described as following sequence: MK1; limonene (4.05 µg·mL^−1^), sabinene (3.20 µg·mL^−1^) and L^−^phellandrene (1.47 µg·mL^−1^), MK2; sabinene (2.55 µg·mL^−1^), terpinen-4-ol (2.05 µg·mL^−1^) and *β*-phellandrene (1.85 µg·mL^−1^), MK3; limonene (6.89 µg·mL^−1^), sabinene (3.00 µg·mL^−1^) and *β*-ocimen (1.47 µg·mL^−1^), HJ; sabinene (4.56 µg·mL^−1^), terpinen-4-ol (4.31 µg·mL^−1^) and γ-terpene (1.08 µg·mL^−1^). To this extend, geographical or environmental factors would play an important role in the chemical composition of the volatiles [12]. The variations due to growing locations of aromatic crops were fully described in chamomile (*Matricaria recutita* L.) [30], *Satureja kitaibelii* [31] and *Myrsine leuconeura* [32]. In the *Zanthoxylum* spp., plants growing at different altitudes yielded essential oil with alternating principal volatiles (limonene, sabinene, and linalool) viz., *Z. armatum* [3,33,34] and *Z*. *alatum* [35]. Our results agree with this as plant samples taken for this experiment were grown at different altitudes.

**Figure 2:**
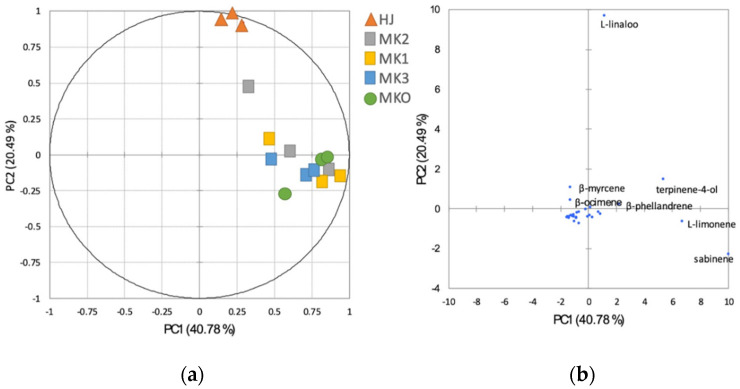
Principal component analysis (PCA) illustrating the relationships among the *Zanthoxylum* species (**a**) and bi-plot factor analysis of the chemical components of the *Zanthoxylum* essential oils (**b**). Abbreviations; HJ (huajiao), MKO (makwoung), MK1 (makhwaen from Mae Tang district), MK2 (makhwaen from Mae Rim district), and MK3 (makhwaen from Song Kwae district).

**Figure 3:**
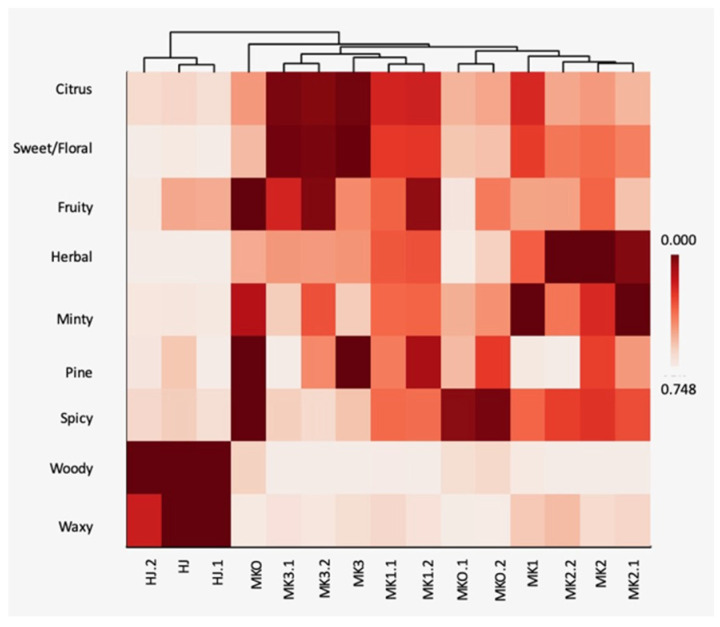
Heatmap relationship of the odor descriptors representing the volatile composition of the *Zanthoxylum* essential oils. Abbreviations; HJ (huajiao), MKO (makwoung), MK1 (makhwaen from Mae Tang district), MK2 (makhwaen from Mae Rim district), and MK3 (makhwaen from Song Kwae district).

The relationships between the chemical components and the *Zanthoxylum* species were analysed using the PCA in Figure 2. The PCA revealed that HJ was distinctive from the other *Zanthoxylum* spp. and MKO could not be detached from MK (PC1 40.78% and PC2 20.49%). According to the bi-plot (Figure 2b), L-linalool was principal in the HJ while L-limonene, terpinen-4-ol, and *β*-phellandrene were among the major components found in MK and MKO. By interpreting the volatile substances according to their descriptors using a heatmap, it was found that HJ was also separated from other species (Figure 3) with different aromatic profile patterns.

Demands of high-quality essential oil from raw material of unique plant taxa for food and perfumery production has ramped up recently. Essential oil compositions could assist in genetic analysis of plant species thus the generic term of chemotypes is well perceived [36,37]. Based on our result of the chemometric analyses, L-linalool was separated from the others and projected with the HJ similar with the result form the RAPD analysis. Therefore, it could be used indirectly as a marker for characterization of the *Zanthoxylum* species. More importantly, the heat mapping of the odor descriptors also convinced that of all the analyzed species, HJ represents the citrusy-floral aroma which is its unique aroma identity. This has been described as the generic perception of Sichuan pepper aroma [38]. Besides, the volatile compositions, non-volatiles such as alkylamides and polyphenols are known as specific chemotypes of the *Zanthoxylum* spp. These compounds offer spice flavor with tingling and numbing sensations [37,38,39].

### 2.5. FTIR Analysis

**Figure 4:**
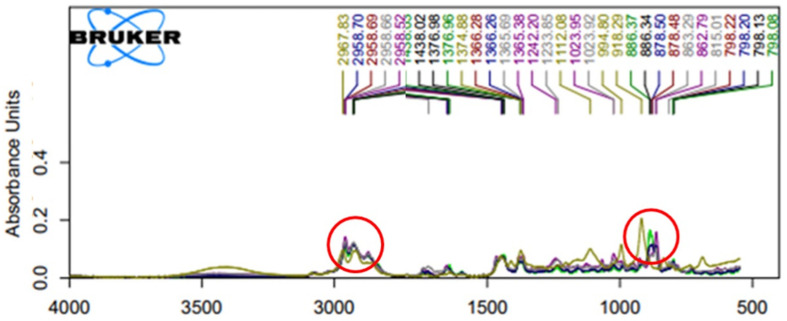
Fourier transform infrared spectrophotometer (FTIR) spectra of the essential oils from five different *Zanthoxylum* species. The insertion is the inset evidence of the peaks between 500–4000 cm^−1^: (---) MK1, (---) MK2, (---) MK3, (---) HJ, and (---) MKO. Abbreviations; huajiao (HJ), makwoung (MKO), MK1 (makhwaen from Mae Tang district), MK2 (makhwaen from Mae Rim district), and MK3 (makhwaen from Song Kwae district).

Fourier transform infrared spectroscopy (FTIR) spectrum patterns have been adopted to expose authentically volatile composition of plant essential oils such as those of lavender (*Lavandula officinalis*), pepper-mint (*Mentha piperita*), green doulas (*Pseudotsuga menziesii*), fir (*Abies alba*), and chicory (*Cichorium intybus*) [40,41]. The spectrum patterns of their EOs responded to the wavenumber ranges 2800–2300 and 1800–1000 cm^−1^) representing of free O-H bond valence and carboxylic acid broadband absorption. Our results illustrated that the oil samples were dominated by overtones and different combinations of C-H reflection and shine occurring between 500–4000 cm^−1^ and aromatic ring at ~1600 cm^−1^. FTIR spectrum scans of the three *Zanthoxylum* species essential oil (MK1-3, HJ, and MKO) absorbed light at a wavenumber range of 1722–798 cm^−1^ and 2967–2926 cm^−1^, respectively, therefore illustrating similar light transmission. EO of the HJ on the other hand showed distinct spectrum characteristics from other samples (Figure 4 and Table 2). This distinction was in parallel with the odor descriptions above analyzed where HJ was indicated to have a sweet and floral scent.

## 3. Materials and Methods

### 3.1. Plant Materials

Three plant specimens of the *Zanthozylum* spp. locally known as makhwaen were collected from the local orchards in three areas: (MK1) Papea, Mae Tang district, Chiang Mai province (19°7′27″ N, 98°42′14″ E); (MK2) Pong Yang, Mae Rim district, Chiang Mai province (18°53′24″ N, 98°49′53″ E); (MK3) Yod, Song Kwae district, Nan province (19°22′37″ N, 100°35′49″ E) in September 2018. Huajiao (HJ) was harvested from Ban Rak Thai, Mok Champae, Muang district, Mae Hong Sorn province (19°32′32″ N, 97°53′35″ E) in September 2018. Makwoung specimen (MKO) was sampled from Phichai, Muang Lampang District, Lampang province (18°22′11″ N, 99°35′44″ E) in September 2018 (Table S3). Based on the samples from harvest, all samples can be divided into two groups: (i) young leaves for DNA analysis and (ii) fruits for essential oil analysis.

The morphological appearances of leaves, flower, and fruit were recorded [19,41]. Their fruits correspondent to all specimen samples were also collected for the essential quality assessment at the mature stage and subjected to the initial drying process as described in the previous report [5]. A taxonomical confirmation has been done by comparison of the taxonomical descriptions with those of the literature data [22] and also confirmed by a botanist. The sample specimens were deposited at Queen Sirikit Botanic Garden (QSBG, Mae Rim, Chiang Mai, Thailand) and the accession numbers of Trid01-05C were assigned.

### 3.2. Morphology Relationship within Species of Zanthoxylum *spp.*

Collected data of the part of Plant for classification were analyzed. Those characters were assigned and scored as plant structure: shrub = 0, tree = 1; thorn: not have thorn = 0, thorn on tree = 1; compound leaf type: odd-pinnate = 0, even-pinnate = 1; number of petals: four petals = 0, five petals = 1, more than six petals = 2; number of anthers: four anthers = 0, five anthers = 1, more than six anthers = 2; fresh fruit color: red = 0, greenish-red = 1, and dry fruit color: brown = 0, no brown = 1. These data were analyzed using cluster analysis (Dendrogram and PCA-biplot) via XLstat, version 2016.

### 3.3. ITS and RAPD Analysis

#### 3.3.1. DNA Extraction

For the extraction of DNA, the DT-S DNA extraction kit (Kurabo, Osaka, Japan) was used with modification of the CTAB extraction procedure. Young leaf tissue of three *Zanthozylum* spp. from five samples (0.5 g) were ground to powder using a mortar and pestle in the presence of liquid nitrogen and transferred to a 1.5 mL polypropylene centrifuge tubes and follow the steps of the DNA extraction kit. Tissue lysis-buffer (MDT) 200 µL and proteinase K (EDT) 20 µL were combined and mixed. After that, the centrifuge tubes were incubated by using the incubator at a temperature of 55 °C for an hour. At this stage, the centrifuge tubes were flipped every 15 min. Then, these tubes were centrifuged at 10,000× *g*. When the process was completed, the supernatant (~200 µL) was moved to a new centrifuge tubes and 180 µL lysis buffer (LDT) was added. Later, these new tubes were centrifuged with vortex for 15 s. before they were incubated at a temperature of 70 °C for 10 min. A solution was moved into the new cartridge tubes and west tubes, then these tubes were aerated. After that, 75 µL wash buffer (WDT) was added into the tubes. These tubes were aerated repeatedly three times to elute DNA. Then, the cartridge tubes were moved into the collection tubes. At this stage, 50 µL elution buffer (CDT) was added and left for 30 min. After that, they were aerated repeatedly for two times. Finally, the centrifuge tubes were tested and stored at a temperature of −20 °C.

After extraction, total DNA was quantified using a nano-drop spectrophotometer (NanoDrop^TM^ 1000 Spectrophotometer, Thermo Fisher Scientific, Bath, UK). For re-quantification, the extracted DNA was run on 1.5% agarose gel electrophoresis using 1× TBE buffer at 5–8 V·mL^−1^ for 30 min and visualized under BLook LED transilluminator (Genedirex, Taoyuan, Taiwan) by staining with MaestroSafe TM (Maestrogen, Las Vegas, NV, USA). The DNA solution was diluted with sterile distilled water (DI) to a concentration of 10 ng·μL^−1^ for PCR analysis and kept at −20 °C until use [42].

#### 3.3.2. ITS Sequence

The ITS2 sequences were amplified using the following pair of universal primers, ITS5-ITS4 (including both ITS1 and ITS2 regions), ITS5 GGAAGTAAAAGTCGTAACAAGG and ITS4 TCCTCCGCTTATTGATATGC. Each 50 µL reaction contained 5 µL 10× PCR buffer, 2.5 µL 2.5 mm MgCl_2_, 0.4 µL 0.2 mm deoxyribonucleotides (dNTP), 5 µL of each primer (10 ng·μL^−1^), 0.4 µL 0.5 U Taq DNA polymerase (HIMEDIA, Mumbai, India), 40 µL sterile distilled water, and 5 µL genomic DNA (50 ng·μL^−1^). The amplification consisted of 94 °C·2 min^−1^, followed by 40 cycles of 94 °C·45 s^−1^, 50 °C·45 s^−1^, and 72 °C·1 min^−1^, and ending with 72 °C for 5 min for the final extension. Amplified products were genotyped using 1.5% agarose gel electrophoresis. Then they were staining with MaestroSafeTM Nucleic Acid Stains (MAESTROGEN, Hsinchu, Taiwan) and visualized under UV transilluminator (BioDoc-It2 imaging systems, Analytik Jena, Thuringia, Germany) before samples were sent to sequencing at Macrogen, Inc. (Seoul, South Korea).

#### 3.3.3. RAPD-PCR Protocols

For RAPD analysis of the genomic DNA, 10-base primers from Operon Technologies (Alameda, GA, USA) and UBC (University of British Columbia, Canada) were chosen (Table S4). A total of nine primers from previous studies were screened [43,44,45,46]. The polymerase chain reaction (PCR) was adjusted to 10 μL^−1^ containing 8 μL^−1^ of OnePCRTM Plus (Genedirex, Taoyuan, Taiwan), 1 μL^−1^ of 1 μm RAPD primer and 1 μL^−1^ of 10 ng genomic DNA. All the reactions were carried out on a Flexcycler2 thermal cycler (Analytik Jena, Thuringia, Germany) using the following profile: 1 cycle, 94 °C, 4 min; 40 cycles, 95 °C, 30 s; 37 °C, 30 s; 72 °C, 60 s; 1 cycle, 72 °C, 10 min. The sample was separated in a 1.5% agarose gel in 1× TBE buffer. The samples were run at 70 V for 120 min. The gels were then visualized using the BLooK LED transilluminator (Genedirex, Taoyuan, Taiwan).

### 3.4. Dendrogram Analysis

The banding pattern for each primer was scored as diallelic (1 = band present, 0 = band absent), and stored in an Excel (Microsoft) spreadsheet file in the form of a binary matrix. To determine the genetic differentiation between the five samples accessions, 10 RAPD markers were analyzed using the statistical package XLSTAT version 2016 software. The coefficients of genetic similarity for all the pair-wise comparisons were computed using Jaccard’s coefficient of similarity and then the distance matrix was subjected to cluster analysis by using the Unweighted Pair Group Method with Arithmetic Mean (UPGMA) to produce a dendrogram.

### 3.5. Essential Oil Analysis

The essential oil was extracted by hydro-distillation for 4 h, from 100 g of dried fruits in 600 mL of DI water in a 2 L flask Clevenger-type apparatus (MTopo^®^, heating mantle, Korea). The oil was dried over anhydrous sodium sulphate (Merck Co., Darmstadt, Germany) and was kept at 4 °C until analysis (usually within three days). The extraction was repeated twice and yield (mean value) was reported as a percentage of essential oil from dry plant material [33].

Gas chromatography-mass spectrometry (GC–MS) analysis was performed on Bruker-Scion 436 GC (Bruker, Hamburg, Germany) a Rxi 5Sil MS (30 m × 0.25 mm; 0.25 μm film thickness) (Restek, Bellefonte, PA, USA). Essential oil samples (2 µL at the dilution of 1%, *v*·*v*^−1^, in dichloromethane (RCI Labscan, Bangkok, Thailand) with a presence of 0.003% *w*·*v*^−1^ toluene (RCI Labscan, Bangkok, Thailand) as an internal standard) were injected in a split mode (1:20). Temperature program includes oven temperature held for 2 min at 60 °C and was enhanced to 150 °C with 3 °C min^−1^. Then, temperature enhancement was programmed up to 270 °C at 5 °C min^−1^ and held at this temperature for 15 min. Other operating conditions include carrier gas was Helium with a flow rate of 1.1 mL min^−1^; injector and detector temperatures were 300 °C, and split ratio, 1:50. Mass spectra (MS), 50–500 (*m*·*z*^−1^) were taken at 70 eV. The mass spectra and retention indices of essential oil components were identified by comparison to MS computer library (NIST 05.L and NIST 98.L. Homologous series of C_8_–C_20_ n–alkanes (Sigma–Aldrich, Steineheim, Germany) were used for identification of all constituents by calculation of the retention indexes (RI). The compounds were confirmed by their RI as well as those from the literature [14]. The amount in µg·mL^−1^ of essential oil was calculated as relative to that of internal standard.

### 3.6. Fourier Transforms Infrared Spectrophotometer (FTIR) Analysis

The FTIR spectrometer used was Bruker model ALPHA II, Diamond ATR (Hamburg, Germany) and operating at the basic of 500–4000 cm^−1^ wavenumbers for averaging 47 scans per spectrum [40].

### 3.7. Statistical Analysis

The data were statistically analyzed using a comparison of the means of yield for essential oils evaluated by Tukey Multiple Comparison’s test at a 95% confidential level. A principal component analysis (PCA) was used to identify the main sources of systematic variation in the chemical compounds data using XLstat software version 2016 [5]. The amount of each volatiles was combined according to their descriptors as described in Sriwichai et al. [6] which then was used to explicate the odor profile of the essential oil. Heatmap was generated with Biovinci software (BioTuring Inc., San Diego, CA, USA).

## 4. Conclusions

Even though a large number of secondary metabolites interfere with DNA sequencing, morphological description is adequate for the differentiation of plant belonging to the *Zanthoxylum* genus. The locally known makhwaen were taxonomically and genetically confirmed as *Z*. *myriacanthum*. From the principal component evaluation, huajiao essential oil was described to have different aroma characteristic as compared to the rest of *Zanthoxylum* spp. analyzed. The essential oils of makwoung and makhwaens from Nan and Chiang Mai were similar in terms of quantity and characteristics of the chemical compositions. For example, limonene and sabinene represent the aroma of citrus and woody. In summary, for sourcing of the raw material, phenotypical characteristic can be used to distinguish the species. Furthermore, the chemical profile of the essential oil depends upon the genotypes which closer similarity was with makwoung and makhwaen, whereas huajiao represented the unique chemotype of citrus-floral aroma.

## Figures and Tables

**Table 1:** Chemical profiles of makhwaen, huajiao, and makwoung essential oils

No.	Chemical Compounds	Descriptors [5]	RI^cal^	RI^ref^	Amount of Chemical (µg·mL^−1^ Essential Oil ^#^)				
					MK1	MK2	MK3	HJ	MKO
**The Amount of Essential Oil Extractions (%)**					7.15 ± 0.07	6.2 ± 0.14	7.4 ± 0.14	5.5 ± 0.01	1.8 ± 0.07
1	α-Thujene	Woody	926	926	0.15 ± 0.01	0.11 ± 0.02	0.04 ± 0.01	0.18 ± 0.02	0.05 ± 0.02
2	α-Pinene	Pine	937	1004	0.42 ± 0.02	0.45 ± 0.02	0.09 ± 0.01	0.52 ± 0.02	0.03 ± 0.02
3	Sabinene ^#^	Woody	942	937	3.20 ± 0.18	2.55 ± 0.02	3.00 ± 0.08	4.56 ± 0.02	0.44 ± 0.02
4	2*β*-Pinene	Pine	974	944	ND	0.02 ± 0.02	ND	0.75 ± 0.02	0.05 ± 0.02
5	*β*-Myrcene	Spicy	993	1132	0.66 ± 0.04	0.27 ± 0.02	0.86 ± 0.05	0.21 ± 0.02	0.13 ± 0.02
6	Octanal	Citrus	999	992	0.06 ± 0.01	0.05 ± 0.02	0.10 ± 0.01	0.02 ± 0.02	ND
7	L-Phellandrene	Fruity	1009	989	1.47 ± 0.07	0.61 ± 0.02	0.32 ± 0.02	0.09 ± 0.02	0.04 ± 0.02
8	Acetic acid, Hexyl ester	Sweet/Floral	1048	1131	0.02 ± 0.01	0.04 ± 0.02	0.02 ± 0.01	ND	ND
9	α-Terpinene	Citrus	1018	1196	0.29 ± 0.02	0.40 ± 0.02	0.08 ± 0.01	ND	ND
10	Methyl (1-methylethyl)-benzene	Citrus	1058	1058	0.16 ± 0.01	0.91 ± 0.02	0.03 ± 0.01	0.50 ± 0.02	0.13 ± 0.02
11	L-Limonene ^#^	Citrus	1047	1035	4.05 ± 0.01	1.01 ± 0.02	6.89 ± 0.18	0.31 ± 0.02	0.97 ± 0.02
12	*β*-Phellandrene	Fruity	1103	1227	1.08 ± 0.08	1.85 ± 0.02	0.90 ± 0.05	0.42 ± 0.02	ND
13	*cis*-Ocimene	Herbal	1132	1132	0.17 ± 0.01	0.11 ± 0.02	0.08 ± 0.01	0.06 ± 0.02	0.02 ± 0.02
14	*β*-Ocimene	Herbal	1144	1017	0.76 ± 0.04	0.31 ± 0.02	1.47 ± 0.06	ND	0.03 ± 0.02
15	γ-Terpinene	Fruity	1168	1168	0.47 ± 0.02	0.63 ± 0.02	0.14 ± 0.01	1.08 ± 0.02	0.25 ± 0.02
16	*trans* Sabinene hydrate	Herbal	1180	1458	0.06 ± 0.01	ND	0.06 ± 0.01	0.39 ± 0.02	ND
17	L-Octanol	Waxy	1167	1578	0.09 ± 0.01	0.17 ± 0.02	0.05 ± 0.01	0.03 ± 0.02	ND
18	α-Terpinolene	Fruity	1236	1236	0.15 ± 0.01	0.17 ± 0.02	0.09 ± 0.01	0.30 ± 0.02	ND
19	L-Linalool	Sweet/Floral	1263	1594	0.43±0.02	0.53 ± 0.02	0.26 ± 0.02	ND	7.35 ± 0.02
20	L-Terpineol	Woody	1189	1387	0.07 ± 0.01	0.11 ± 0.02	0.09 ± 0.07	0.21 ± 0.02	0.08 ± 0.02
21	Terpinen-4-ol	Citrus	1391	1566	1.09 ± 0.07	2.05 ± 0.02	0.39 ± 0.03	4.31 ± 0.02	0.14 ± 0.02
22	Sabina ketone	Minty	1236	1161	0.03 ± 0.01	0.06 ± 0.02	ND	ND	ND
23	*β*-Thujone	Minty	1251	1011	ND	ND	ND	ND	1.03 ± 0.02
24	*β*-Fenchyl alcohol	Pine	1263	1130	0.37 ± 0.08	0.53 ± 0.02	0.13 ± 0.08	0.27 ± 0.02	0.16 ± 0.02
25	*trans*-Piperitol	Herbal	1282	1210	0.02 ± 0.02	ND	ND	0.12 ± 0.02	ND
26	Decanal	Sweet/Floral	1423	1423	0.22 ± 0.02	0.16 ± 0.02	0.24 ± 0.01	0.17 ± 0.02	ND
27	Acetic acid, 2-Ethylhexyl ester	Herbal	1370	-	0.28 ± 0.01	0.48 ± 0.02	0.25 ± 0.02	ND	ND
28	*trans*-Geraniol	Sweet/Floral	1442	1442	0.03 ± 0.01	0.12 ± 0.02	ND	ND	0.55 ± 0.02
29	1-Decanol	Sweet/Floral	1457	1457	0.03 ± 0.03	0.07 ± 0.02	0.04 ± 0.01	0.06 ± 0.02	ND
30	2-Undecanone	Fruity	1467	1467	0.47 ± 0.02	0.04 ± 0.02	0.36 ± 0.04	ND	ND
31	Geranyl acetate	Sweet/Floral	1408	1408	0.26 ± 0.01	0.23 ± 0.02	0.03 ± 0.01	ND	ND
32	Dodecanal	Citrus	1439	1439	0.07 ± 0.01	0.05 ± 0.02	0.06 ± 0.01	ND	ND
33	*trans*-Caryophyllene	Spicy	1441	1441	0.03 ± 0.03	0.02 ± 0.02	0.03 ± 0.01	0.09 ± 0.02	ND
34	D-Germacrene	Woody	1447	1447	0.09 ± 0.01	ND	ND	0.03 ± 0.02	ND
35	Bicyclogermacrene	Woody	1457	1457	0.02 ± 0.02	ND	ND	ND	ND
	Total			-	16.76	13.09	16.09	14.68	11.44

RI^cal^: Calculated retention index. RI^Ref^: Retention index from the referent [5]. ^#^ Values are calculated as a reference to internal standard toluene (0.003% *w*·*v*^−1^). Makhwaen fruit, huajiao and makwhoung essential oil were analyzed by GC-MS (MK1, MK2, MK3, HJ, and MKO). ND: not detected.

**Table 2:** Wavenumbers and functional groups of *Zanthoxylum* spp. essential oils.

Name	Wavenumber (cm^−^^1^)	Type of Vibration	Functional Groups
HJ	918.29	(=C-H) bending strong	Alkene
	994.8	(=C-H) bending strong	Alkene
	1112.08	C-O stretch strong	Alcohol
	1374.88	bending variable -C-H	Alkane
	2967.83	C-H stretch strong	CH_2_ group
MKO	862.79	(=C-H) bending strong	Alkene
	1023.95	C-O stretch strong	Alcohol
	1365.38	bending variable -C-H	Alkane
	1445.39	bending variable -C-H	Alkane
	2958.52	C-H stretch strong	CH_2_ group
MK1	878.5	(=C-H) bending strong	Alkene
	1366.26	bending variable -C-H	Alkane
	1445.3	bending variable -C-H	Alkane
	1601.0	-	Aromatic ring
	1650.64	(C=O) stretch	Ester
	2958.7	C-H stretch strong	CH_2_ group
MK2	863.29	(=C-H) bending strong	Alkene
	1233.85	(C-O) stretch	Alcohol
	1365.69	bending variable -C-H	Alkane
	1446.33	bending variable -C-H	Alkane
	2958.66	C-H stretch strong	CH_2_ group
MK3	886.37	(=C-H) bending strong	Alkene
	1376.96	bending variable -C-H	Alkane
	1438.03	bending variable -C-H	Alkane
	1564.0	-	Aromatic ring
	1643.61	(C=O) stretch	Alcohol
	2926.69	C-H stretch strong	CH_2_ group

Abbreviations: huajiao (HJ), makwoung (MKO), and makhwaen (MK1-3).

## Data Availability

Not applicable.

## Supplementary Materials

The following are available online at https://www.mdpi.com/article/10.3390/plants10040803/s1, Figure S1: The dendrogram of *Zanthoxylum* spp. in North of Thailand; huajiao (HJ), makwhoung (MKO), MK1 (makhwaen from Mae Tang district), MK2 (makhwaen from Mae Rim district) and MK3 (makhwaen from Song Kwae district) derived by UPGMA from the similarity matrix based on seven morphology data (plant structure, thorn, compound leaf type, petals, anthers, fresh and dry fruit color); Table S1: Plant characteristics for taxonomical identification of collected *Zanthoxylum* spps. used in this experiment; Table S2: Floral and fruit characteristics of makhwaen collected from different locations (MK1-3); Table S3: Study site the sample collections; Table S4: Sequence of RAPD primers.
