# Supplementary material for: Aromatic Profile Variation of Essential Oil from Dried Makwhaen Fruit and Related Species
Source: Plants (Basel). 2021 Apr 19;10(4):803. doi: 10.3390/plants10040803 (PMC8072721; doi:10.3390/plants10040803)
Supplement: Supplementary file 1 [file plants-10-00803-s001.zip › plants-1175898-supplementary.pdf]

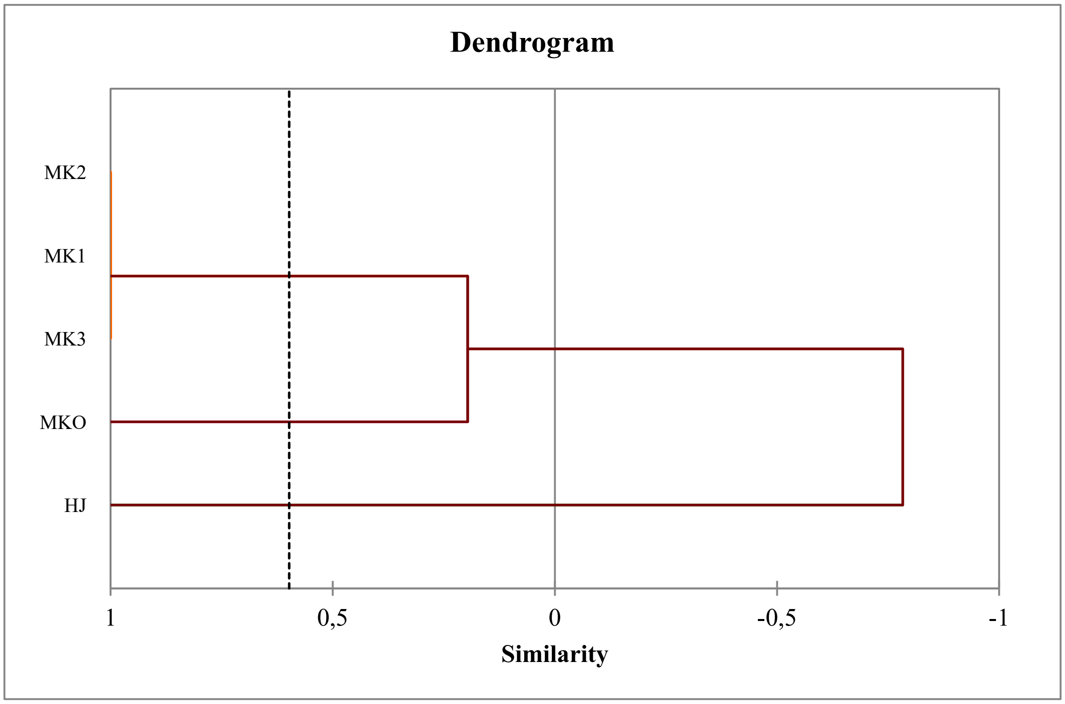

Figure S1. The dendrogram of *Zanthoxylum* spp. in North of Thailand; huajiao (HJ), makwhoung (MKO), MK1 (makhwaen from Mae Tang district), MK2 (makhwaen from Mae Rim district) and MK3 (makhwaen from Song Kwae district) derived by UPGMA from the similarity matrix based on seven morphology data (plant structure, thorn, compound leaf type, petals, anthers, fresh and dry fruit colour).

Table S1

Plant characteristics for taxonomical identification of collected *Zanthoxylum* spps. used in this experiment

	Common names		
**Part of Plant for classification**	**huajiao (HJ)**	**makwoung (MKO)**	**makhwaen (MK1-3)**
**Plant structure**	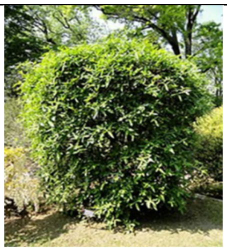<br>Shrub/*1	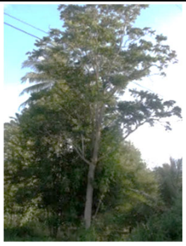<br>Tree	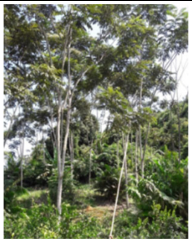<br>Tree
**Thorn**	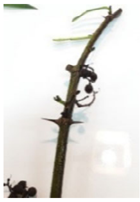<br>Thorn on leaves	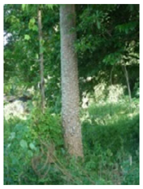<br>Thorn on tree	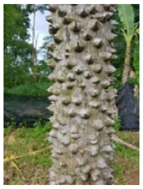<br>Thorn on tree
**Leaf type**	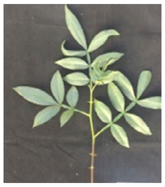<br>Odd-pinately compound leaf	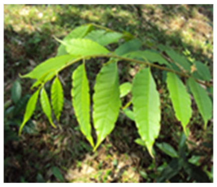<br>Even -pinately compound leaf	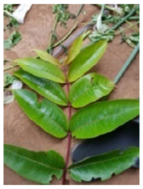<br>Even-pinately compound leaf
**Number of petals**	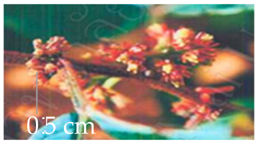<br>6-9 petals	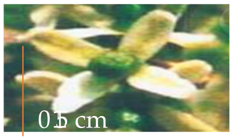<br>4 petals	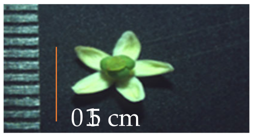<br>5 petals
**Number of anthers**	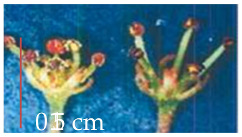<br>4-8 anthers	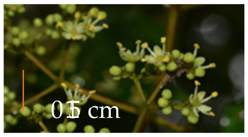<br>3-4 anthers	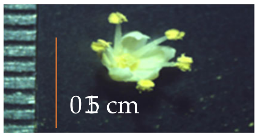<br>5 anthers
**Fresh fruit colour**	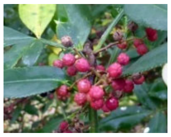	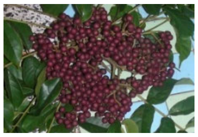	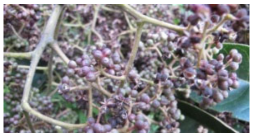

	Red*[2]	Red	Greenish red
**Dry fruit colour**	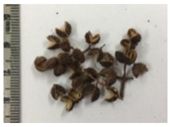<br>Brown	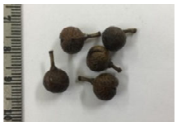<br>Dark brown	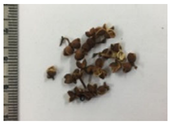<br>Brown
**Scientific name****	*Zanthozylum armatum*	*Zanthozylum rhetsa*	*Zanthozylum myriacanthum*

[*1] online; https://en.wikipedia.org/wiki/*Zanthoxylum_armatum*

[2] online; https://www.flickr.com/photos/helicongus/15771073680

** the specimens were taxonomical confirmed by Queen Sirikit Botanic Garden, Chiang Mai, Thailand and as in Sriwichai *et al.,* [5].

Table S2

Floral and fruit characteristics of makhwaen collected from different locations (MK1-3)

Part of Plant for classification	makhwaen (MK1)	makhwaen (MK2)	makhwaen (MK3)
**Number of petals**	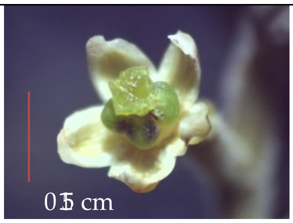<br>5 petals	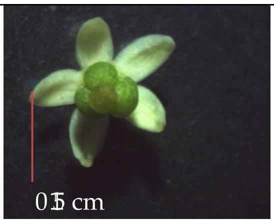<br>5 petals	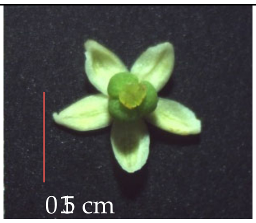<br>5 petals
**Number of anthers**	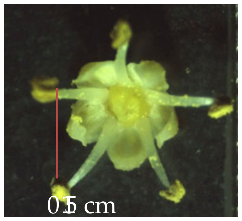<br>5 anthers	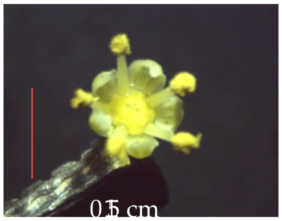<br>5 anthers	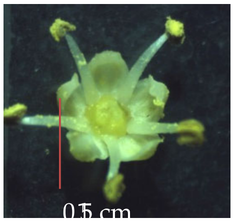<br>5 anthers
**Fruit structure**	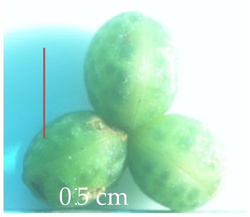<br>3 capsules	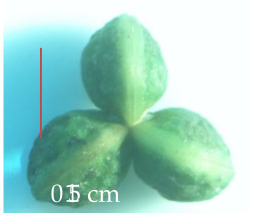<br>3 capsules	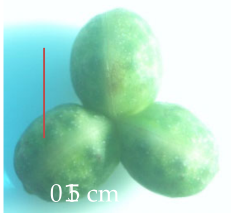<br>3 capsules

Table S3

Study site the sample collections

Location	Coordinate	Altitude (m)	Picture of area
Papea, Mae Tang district, Chiang Mai province (MK1)	19° 7' 27’’N, 98° 42' 14’’ E	924	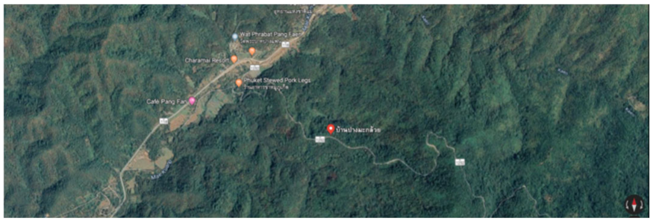
Pong Yang, Mae Rim district Chiang Mai province, (MK2)	18° 53' 24’’N, 98° 49' 53’’E	800	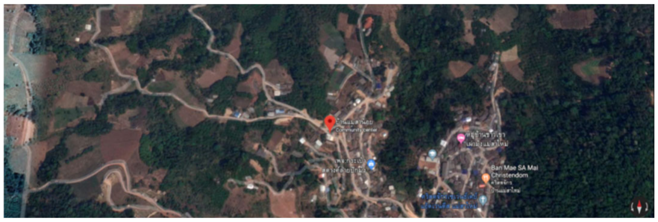
Yod, Song Kwae district, Nan province (MK3)	19° 22' 37’’N, 100° 35' 49’’E	1,600	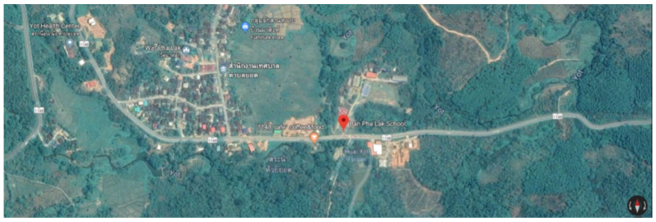
Ban rak Thai Mok Champae, Moung district, Mae Hong Sorn province (HJ)	19° 32' 32’’N, 97° 53' 35.’’E	1,176	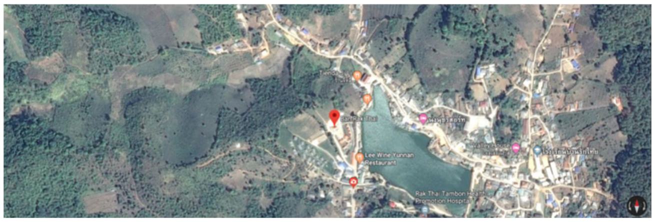
Phichai, Mueang Lampang district, Lampang province (MKO)	18° 22' 11’’N, 99° 35' 44’’E	294	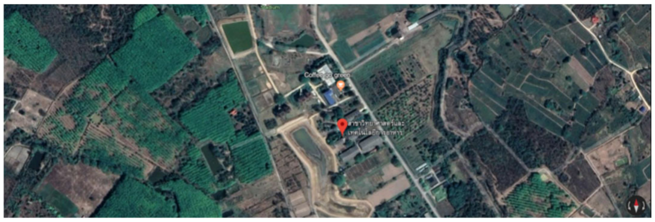

* The points of sampling location are presented in red pin.** Satellite images by Google Maps

Table S4

Sequence of RAPD primers

Primer	Sequence	References
**S6**	TGCTCTGCCCC	[43]
**S7**	GGTGACGCAG	[43]
**S9**	GCGTCGAGGG	[43]
**OPA01**	CAGGCCCTTC	[44]
**OPA04**	AATCGGGCTG	[44]
**OPN05**	AGGGGTCTTG	[45]
**OPN06**	GAGACGCACA	[45]
**OPN07**	CAGCCCAGAG	[45])
**OPN13**	AGCGTCACTC	[46]
